# Understanding the effects of COVID-19 stigmatisation on job performance: a survey of frontline healthcare workers

**DOI:** 10.1080/07853890.2022.2089910

**Published:** 2022-08-16

**Authors:** Sabina Ampon-Wireko, Lulin Zhou, Prince Ewudzie Quansah, Ebenezer Larnyo

**Affiliations:** School of Management, Jiangsu University, Zhenjiang, China

**Keywords:** COVID-19 stigmatisation, resilience, anxiety, job performance, frontline health workers

## Abstract

The level of stigmatisation among health care providers has increased during the COVID-19 pandemic, and understanding the effect of COVID-19 stigmatisation on job performance has become increasingly important. The study explores the influence of COVID-19 stigmatisation on job performance among frontline health workers *via* the mediating role of anxiety. Furthermore, the moderating effect of resilience in the association between COVID-19 stigmatisation and anxiety is further examined. Participants were made up of 820 frontline health workers working in the epicentres of the Bono Ahafo, Western, Greater Accra, and Northern regions of Ghana. The hierarchical regression technique was employed in estimating the relationship between the variables. COVID-19 stigmatisation among frontline health workers directly affected anxiety and performance. In addition, the results showed that resilience moderated the relationship between COVID-19 stigmatisation and anxiety. The findings again demonstrated that anxiety partially mediated the association between concern for disclosure and public attitude and negative experience and job performance, whereas personalised stigma was insignificant. The study provides implications for establishing anti-stigma interventions and programs to enhance job performance among health workers.

**Key messages**
1. Many healthcare workers are subject to stigmatisation during the COVID-19 pandemic.
2. The study employs hierarchical regression methods to examine the impacts of COVID-19 stigmatisation on job performance among frontline health workers.
3. The health management team should strengthen interventions to control the stigma experienced by health workers during COVID-19 treatments.

## Introduction

The COVID-19 disease caused by the novel beta-coronavirus has resulted in severe consequences and unparalleled levels of misery and unemployment. The pandemic has undoubtedly caused a deep global recession and uncertainty [1]. Throughout the pandemic, people's attitudes regarding frontline health workers (FHW) have shifted. Frontline health workers in this present study refer to all types of health workers, including nurses, community health workers, pharmacists, doctors, midwives, etcetera, who directly treat people afflicted with the COVID-19 virus. While they were frequently glorified as heroes in the first wave, the public's opinion turned against them during the second wave [2], and the intricacy of the COVID-19 stigmatisation in Ghana is quite alarming [3]. The reason could be that COVID-19 is a novel disease, and the uncertainty could exacerbate community anxiety [4,5].

With the third wave, more than a year after the pandemic, social isolation has reduced, but this has not immediately benefitted the workers' mental health [2]. The stigmatisation of health care behaviours has led to increased malpractice complaints [6]. The psychological implications of the COVID-19 epidemics are still being investigated worldwide. Some studies link stigma to workers' mental health during the pandemic [7,8]. Studies in Italy, China, and the United States found symptomatology for generalised anxiety disorder and post-traumatic stress disorder, particularly among healthcare workers during the COVID-19 pandemic [9,10]. Liu et al. [11] looked at the state anxiety and trait anxiety related to the COVID-19 pandemic. The study found that the ratio of state anxiety in respondents was more significant than the ratio of trait anxiety, an indication that the COVID-19 pandemic could play a role in causing anxiety. The present study moved further to study the effects of COVID-19 stigmatisation on healthcare personnel's anxiety and job performance.

The stigma experienced by the healthcare personnel who manage HIV/AIDS/tuberculosis patients [12,13] and Ebola patients have been studied [14] with less focus on the stigma associated with health workers treating COVID-19 patients, and this happens to be a crucial objective of this study. To justify why the current research is critical and what it adds to what is currently known about healthcare employees, we examined the effects of COVID-19 stigmatisation on job performance based on the [15] theory of stigmatisation. This study aims to find out whether resilience moderates the link between COVID-19 stigmatisation and anxiety and assess if COVID-19 stigmatisation impacts job performance *via* the mediating effects of anxiety among healthcare personnel who play a major role in addressing COVID-19 outbreaks. The findings of this study will enable the health management team to provide health policies that will eliminate the COVID-19 stigma to enhance performance among health workers.

## Methods

### Type of research, study settings, and sampling

A descriptive cross-sectional study was employed in this study. Frontline healthcare professionals working within the epicentres of the Bono Ahafo, Western, Greater Accra, and Northern regions with at least six months of patient care experience were chosen for the study. In selecting an adequate and appropriate sample size for this current study, we relied on the variable-to-sample ratio [16,17].

The variable to-sample ratio suggests that a proposed sample size selection should be based on the ratio of respondents to items [17]. The ratio is expressed as N: p. The N represents the number of respondents while the p represents the number of items. Sample suggestions for the variable to item ratio include 3:1, 6:1, 15:1, and even 20:1. However, we employed the 10: 1 ratio for this research work. This ratio means that ten respondents to an item each was used following the suggestions of other previous studies like Cattell [18] and Kline [19]. Considering the total thirty-five items used in assessing the study’s variables, we could have settled on 350 respondents.

However, the current study collected 549 valid responses from frontline healthcare professionals. The 549 valid responses exceed the 350 respondents; hence, this current study’s data is very sufficient to carry out any further analysis. In addition, we used the purposive sampling technique in selecting the respondents. It is robust to permit a researcher to gather data from a convenient and accessible element of a population. We, therefore, used a sample size of 820, out of which participants with missing and non-response data were deleted from the specific statistical models. Unfortunately, there are no perfect solutions to this problem [20]; hence the researchers selected one of several advanced procedures (deletion) that increases estimated parameter accuracy while avoiding error inflation.

### Theoretical background and hypothesis development

Stigmatisation stems from adverse outcomes such as devalued social identities, prejudice, stereotyping, discrimination, and neglect [21,22]. Goffman's theory, also popularly known as the dramaturgical approach, has been proposed to explain how stigmatisation affects workplace outcomes. Relying on the Goffman theory, it is posited that frontline health workers, exposed to COVID-19 stigma cannot meet everyday work expectations and may perform poorly at work.

Kreiner *et al.* [23] addressed the issue of stigma in the contexts of AIDS and cancer, and similar research in the context of COVID-19 is needed. Based on the Goffman theory of stigmatisation, it is expected that frontline health workers who are stigmatised due to their association with COVID-19 patients would experience anxiety, which will influence their job performance. It is again theorised that increasing resilience will reduce the relationship between COVID-19 stigma and anxiety.

**Figure 1.**
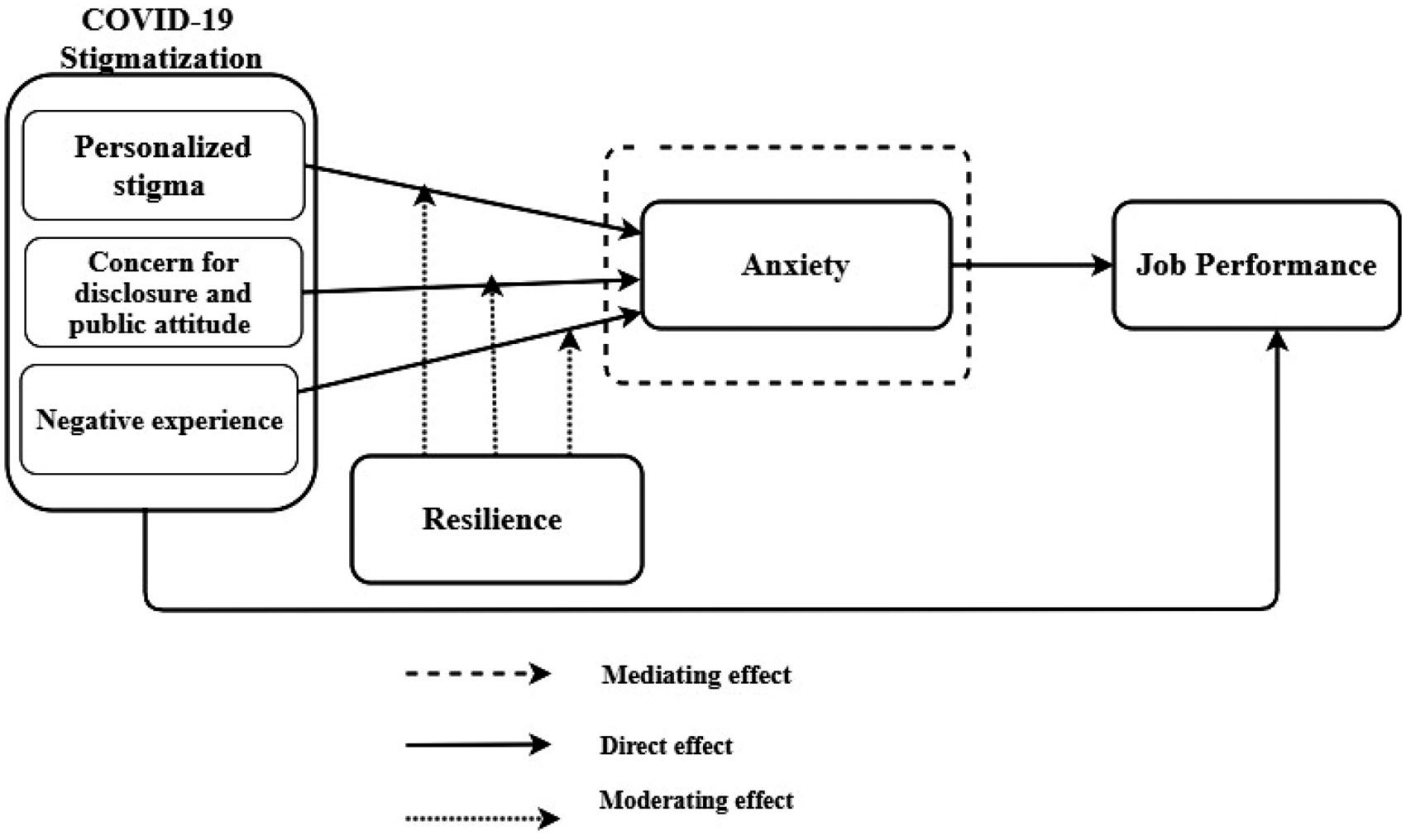
Conceptual framework.

For these reasons, the current study points to the need to explore the impacts of stigma on frontline health workers' job performance during COVID-19. This will improve understanding and suggest paths for stigma management for many frontline health workers suffering from COVID-19 stigmatisation in Ghana, and Figure 1 provides the conceptual framework of the study.

### Stigmatisation, anxiety, and job performance

Motowidlo [24] described job performance (JP) as the forecasted benefit from an employee's actions over a given time frame. It determines whether or not an individual does a good job [25]. Numerous researchers have examined health workers' performance [26–28]. It is believed that investigating the effects of stigmatisation on JP on FHWs using Gofman's theory could guide decision-makers and researchers to augment performance among healthcare professionals in Ghana.

The COVID-19 pandemic issue has changed the work atmosphere, and employment needs dramatically. A survey of 1210 people from the general population in China during the early stages of the COVID-19 infection found that more than half of the people had moderate to severe psychological effects [29].

Ramaci et al. [30] reported that health care employees who manage COVID-19 patients experience stigma, damaging their ability to execute their jobs.

Other researchers [31,32] have also explored the effects of stigma on anxiety. Some hospital staff assessments found that HCWs reported anxiety symptoms, with concerns centred on the viral infection, the fear of infecting families, and the associated health consequences [33,34]. This research revealed that coming into contact with infected individuals can increase infection-related anxiety and symptoms of psychosomatic tiredness [33].

Teksin et al. [35] established that the perception of stigma score was significantly higher among HCWs who had worked with patients with COVID-19. They also found a positive correlation between stigmatisation and anxiety using the multicenter study in Turkey. A multivariable logistic regression analysis [36] in Nepal established that stigma faced by health workers was significantly associated with higher odds of experiencing symptoms of anxiety. Akdağ et al. [37] established that as stigma increased, depression and anxiety symptoms in Turkey increased. Per the previous literature, it is hypothesised that:

Hypothesis 1: Stigmatisation will significantly relate to job performance

Hypothesis 1a: There is a significant negative relationship between personalised stigma and job performance

Hypothesis 1b: Concern for disclosure and public attitude will significantly relate to job performance

Hypothesis 1c: There is a significant negative relationship between negative experience and job performance

Hypothesis 2: Stigmatisation will significantly relate to anxiety

Hypothesis 2a: There is a significant negative relationship between personalised stigma and anxiety

Hypothesis 2b: Concern for disclosure and public attitude will significantly relate to anxiety

Hypothesis 2c: There is a significant negative relationship between negative experience and anxiety

### Anxiety as a mediator between stigmatisation and performance

Anxiety is common in unexpected situations, such as the pandemic that is currently occurring [38]. In the context of the COVID-19 pandemic, Liu et al. [11] study discovered that the epidemic might be contributing to anxiety. This provides an opportunity to investigate the impact of COVID-19 stigma on healthcare workers' anxiety and performance. Chen et al. [39] discovered a significant connection between stigma and anxiety in family care. Correspondingly, anxiety and depression scores were positively correlated with stigma perception among HCWs. However, in China, individual nurses refused any psychological help. They denied any problems despite showing unwillingness to rest, and signs of psychological distress [40] in schizophrenia patients. Using structural equation modelling, Yeni et al. [41] established a significant positive association between stigma and anxiety.

Recent research has demonstrated the relevance of anxiety in the workplace. For example, employees anxious about meeting their employer's expectations are less productive at work [42,43]. Anxiety's energy effect impairs employees' ability to perform their job duties [44]. Additionally, employees who experience significant anxiety at work are likely to be dissatisfied with Chen et al. [45], further impairing their job performance.

Similarly, workers may interpret their experience of anxiety as a signal that their organisation is unconcerned about their physical well-being [46], which contributes to negative perceptions of the employer and decreases employees' willingness to perform job duties that would otherwise affect organisational effectiveness. De Clercq et al. [47] established self-efficacy has a significant indirect effect on job performance *via* job-related anxiety. The current study believes that anxiety can help explain the stigma's impact on job performance. We believe that stigmatising frontline health workers may increase job-related anxiety, reducing job performance. Employees who engage in inappropriate behaviours such as worrying and agonising could deplete their energy for productive behaviours. With the existence of such research streams and empirical findings elsewhere, this study proposes the following hypothesis:

Hypothesis 3: Anxiety will act as a mediator between stigmatisation and job performance

Hypothesis 3a: Anxiety will significantly mediate the relationship between personalised stigma and job performance

Hypothesis 3b: Anxiety will significantly mediate the relationship between concern for disclosure, and public attitude will significantly relate to job performance

Hypothesis 3c: Anxiety will significantly mediate the relationship between negative experiences and job performance

### Resilience as a moderator between stigmatisation and anxiety

As nations suffer a common stressor: the COVID-19 pandemic, anxiety and resilience have become more critical. According to a study, resilience can prevent mental illnesses like anxiety and despair [48]. This suggests that resilience is linked to health care workers' anxiety levels, in the sense that the greater resilient a person gets, the better their mental health. According to Mahmood and Ghaffar [49], resilience is a good adaptability process in stressful situations. When it comes to job productivity, stigmatised workers cannot meet daily work demands [50] and report lower job satisfaction, performance, job involvement, and desire to learn and develop.

According to Gheshlagh et al. [48], resilience is associated with health care providers' anxiety levels because the more resilient a person becomes, the healthier their mental health becomes. The transitional model described by Garmezy et al. [51] established resilience acts as a moderator, mitigating the detrimental influence of threats on the development of psychosocial function. According to Ifeagwazi et al. [52], resilience moderates socioeconomic estrangement and psychological suffering. Yi et al. [53] demonstrated that participants with low resilience exhibited fewer self-care actions as anxiety levels increased. Regrettably, there are no published studies on the role of resilience in the association between stigma and anxiety among healthcare providers. On the ground of prior investigations, it is hypothesised;

Hypothesis 4: Resilience will play a moderating role in the relationship between stigmatisation and job performance

Hypothesis 4a: Resilience will significantly moderate the relationship between personalised stigma and anxiety

Hypothesis 4b: Resilience will significantly moderate the relationship between concern for disclosure and public attitude and anxiety

Hypothesis 4c: Resilience will significantly moderate the relationship between negative experiences and anxiety

### Study data collection

The study design was written per the Strengthening reporting of Observational Studies in Epidemiology (STROBE) protocol [54]. The researchers approached the respondents consensually and meticulously after obtaining permission from the health management team to warrant anonymity and confidentiality. Health managers who worked in the isolation wards but were not directly involved in the care of COVID-19 patients were excluded from the study. Health authorities recommended the first participant willing to share their COVID-19 stigma experience, and then a snowball sampling technique was used to trace additional participants. The consent form also included the eligibility requirements.

The eligibility included prospective participants answering yes to the following two questions: (1) Have you assisted in treating COVID-19 patients? Yes, or No? (2) Will you be willing to participate in three data collection waves performed at nearly one-month time intervals? Yes or No? The study took place from September 2020 to January 2021.

The respondents agreed to participate in the research without compensation and completed the questionnaire anonymously. The research complies with the 1995 Declaration of Helsinki (as revised in Edinburgh in 2000). All applicable ethical guidelines for conducting human research were followed, including compliance with Ghana’s legal requirements. No treatment was administered to the participants, including intrusive diagnostics, or procedures having caused psychological or social discomfort; thus, no additional ethical clearance was obliged.

As a result, the questionnaires were distributed to 820 frontline health workers in the first wave of data collection. In all, we were able to collect 573 questionnaires that matched. Participants received surveys with reply envelopes in their email due to the ongoing COVID-19 disease epidemic. The participant's socio-demographic variables were assessed in the first section of the questionnaire. The main variables, such as stigmatisation, resilience, anxiety, and job performance, are discussed in the next section.

This study employed both multi-wave and multi-source survey research designs for the data collection. The objective of the multi-wave and multi-sources survey was to minimise common method bias. We collected data in three different waves, with a one-month gap between each wave of data collection. After many reminders and confidentiality assurances, we obtained 820 frontline health professionals who agreed to participate in our data collection in the first wave. This contained items about the COVID-19 stigmatisation of our respondents, out of which 711 returned their questionnaires. During the second wave of data collection, we distributed items on "resilience" to 711 respondents who returned their questionnaire in the first wave because some respondents had travelled to the regional capital for a workshop. A total of 649 respondents returned their questionnaires in the second wave.

In the third wave, the questionnaire containing the items on demographic characteristics and anxiety to the 661 respondents was distributed, out of which 631 respondents returned their questionnaires. We were informed that some health workers had been transferred to different regions, and some were on study leave. Five hundred and sixty-one (561) respondents returned their questionnaires in the third wave. In all, 549 valid responses matched in the three waves. To collect data on the job performance, the participant's managers were asked to rate the performance of the respondents. There were 134 managers that assessed the performance of 549 participants.

According to the administration and structure of the hospitals, the number of nurses who reported to single-line managers ranged from one to eight. In this research work, the number of participants for each line manager ranged from 1 (minimum) to 5 (maximum). The average age of the respondents was 39, and they had an average of 7 years of professional experience.

## Survey instrument

### Job performance (JP)

We described "work performance" as the ability of employees to complete tasks and meet organisational goals and the outcomes of such accomplishments [55]. We measured JP with four items adapted from the work of Baird [56]. Sample items include "How do you evaluate your work effort?" and "How do you evaluate the quantity of your work?" These items have been used in previous studies and recorded high internal consistency values greater than 0. For instance, Ang et al. [57] recorded a Cronbach alpha value of 0.893 to establish the reliability of the job performance scale. Song and Chathoth [58] also had the Cronbach alpha value to be 0.861 in their study. In this current study, the Cronbach alpha value for the job performance scale is 0.926 and is even better than in previous studies. Items for JP were rated on a 7-point Likert scale ranging from 1 (extremely low) to 7 (extremely high).

### COVID-19 stigmatisation

The COVID-19 stigmatisation scale is made up of sixteen (16) items categorised into three subscales: personalised stigma (PES), concern for disclosure and public attitude (CDP), and negative experience (NEX) [59]. **PES** items have received high recognition and acceptance in previous studies. Examples of such items include “People don’t want me around their children once they know I am an FHW.” and “People have physically backed away from me when they learn I am an FHW.” The Cronbach's α coefficient based on the previous research [59] was 0.907, and in this current study, the Cronbach alpha 0.96 indicates high internal consistency.

By assessing the CDP construct, we employed five items from [59]. Sample items include, “Telling someone I am an FHW is risky" and "I am very careful who I tell that I am an FHW." The Cronbach alpha for CDP in the current study is 0.912. Items measuring the NEX construct had high reliability in previous studies with Cronbach alpha 0.789. Sample items comprise “I regret having told some people that I am an FHW” and “I have been hurt by how people reacted to learning I am an FHW.” All COVID-19 stigma items were rated on a seven-point Likert scale from one (strongly disagree) to seven (strongly agree).

### Resilience (RES)

The 10-item Connor-Davidson Resilience Scale (CD-RISC10) was extracted from the original 25-item Connor-Davidson Resilience Scale (CD-RISC), which is a self-rated instrument to measure resilience [60] following [39]. This five-point scale measures resilience on a scale of 1 to 5, with higher scores indicating increased resilience. Researchers widely use the CD-RISC10 due to its high internal consistency and reliability of the constructs [39,61].

### Anxiety (ANX)

To assess job-related anxiety, we used five items from the [62] scale, which has been used in other studies [63,64]. The respondents indicated, for example, whether they had experienced "nervousness" as a result of their job and whether they felt "guilty" when they took time off from work. Anxiety items were rated on a five-point Likert anchors scale ranging from 1 (extremely low) to 5 (extremely high). Cronbach alpha 0.789 was highly reliable for items measuring the ANX construct in previous studies [47].

### Control variables

The variables such as gender, age, education, and marriage were employed as controls during the hierarchical regression analysis. We choose the variables because they have been identified by [65] to influence performance among health workers.

### Data analysis

Preliminary analysis was performed using Statistical Package for the Analysis of a Moment Structure (AMOS) version 24 and Social Science (SPSS) v. 26.0 and software for testing the hypothesised relationships. A hierarchical regression analysis was used to estimate the various hypothesised relationships depicted in the conceptual framework. We firstly tested for the impact of COVID-19 stigmatisation on job performance. Next, the mediation anxiety in the relationship between COVID-19 stigma and job performance was assessed. Finally, to determine resilience's principal and moderating effects on anxiety (Figure 1), we centralised COVID-19 stigmatisation (personalised stigma; concern for disclosure and public attitude and negative experience) and anxiety to overcome multicollinearity issues. The study introduced the demographic variables into the regression equation to restrict their impact on anxiety in the first stage. In the second stage, personalised stigma (PES) was entered into the regression equation to predict anxiety for frontline health workers. During the third stage, resilience was introduced into the model. In the fourth stage, the study incorporated the interaction term (PES × resilience) into the model.

### Common method bias test

We employed various steps to handle common method variance in our data. While designing and distributing the questionnaires, we followed the proposed steps of Podsakoff et al. [66]. The steps included randomising the items' order and issuing reports to the respondents that the research was solely for academic purposes. Also, we informed the respondents that they should feel free to choose any answer they deemed fit and that there was no right or wrong answer. Furthermore, P Podsakoff et al. [66]; Podsakoff et al. [67] highlight that participants are more motivated to be more accurate if they believe the information provided will benefit the organisation, and favourable feedback may also motivate greater accuracy. For this reason, we assured the respondents that the information they provided would enable the design of specific policy guidelines to encourage management support, increase clear motivation and achieve high job satisfaction.

Again, we kept the survey items short to minimise redundant measures and overlap, which helped the participants give more accurate responses. Respondents assured that their responses would remain anonymous to alleviate assessment concerns and social desires. According to [68], this process assists respondents in projecting an objective image of themselves. We further employed Harman’s one-factor test to identify threats of common method bias. An unrotated, principal component factor examination of all measurement items showed six factors with eigenvalues above one. The first factor explained 24.39% of the total variance, less than 50%, while all elements explained 77.25%.

## Results and discussion

### Measurement model, construct validity, and reliability

In conformity with standard practice in literature and to ensure that the variables achieved greater acceptability for further analysis, we subjected the data to validity and reliability testing with SPSS version 23 software. We performed an exploratory factor analysis (EFA) to see if the items for the survey could load onto their predicted variables since some modifications were made to the original items. All items were loaded onto their corresponding constructs and recorded factor loadings greater than 0.50. The SPSS was also used to check the reliability of the scales. All the scales had Cronbach alpha (*α*) coefficient values above the proposed 0.70 thresholds in previous studies like Nunnally [69], Jöreskog et al. [70], and Kline [71]. The reliability in Table 1 shows that the scales had high internal consistencies.

**Table 1.** Result of the confirmatory factor analysis and reliability testing.

	Factor loading	Cronbach alpha	AVE	CR
RESI2	0.928	0.98	0.835	0.981
RESI3	0.927			
RESI6	0.921			
RESI5	0.921			
RESI7	0.919			
RESI10	0.91			
RESI1	0.908			
RESI4	0.907			
RESI9	0.903			
RESI8	0.892			
PES6	0.903	0.96	0.981	0.961
PES7	0.897			
PES5	0.887			
PES8	0.885			
PES4	0.875			
PES3	0.874			
PES2	0.87			
PES1	0.746			
CDP4	0.835	0.912	0.638	0.898
CDP3	0.817			
CDP5	0.795			
CDP1	0.795			
CDP2	0.748			
PER2	0.826	0.926	0.653	0.883
PER3	0.812			
PER4	0.801			
PER1	0.794			
ANX4	0.775	0.721	0.583	0.875
ANXI2	0.767			
ANXI3	0.764			
ANXI1	0.716			
ANX5	0.794			
NEX2	0.803	0.84	0.600	0.818
NEX1	0.786			
NEX3	0.733			

Abbreviations: RESI, Resilience; PES, Personalised stigma; CDP, Concern for disclosure and public attitude; PER, Performance; ANX, Anxiety; and NEX, negative experience.

Furthermore, we performed a validity test with critical interests in standardised factor loadings, fit indices, average variance extracted (AVE), composite reliability (CR), and discriminant validity. The standardised factor loadings for all the variables presented in Table 2 ranged from 0.716 to 0.928. A critical look at the factor loadings shows all the items are more significant than 0.70. The values of Cronbach are all greater than 0.7. Also, the composite reliability (CR ranged from 0.721 to 0.98, and AVEs (ranged from 0.600 to 0.981) for the variables satisfy the recommended threshold of 0.50 or higher, respectively. These outcomes are indications that the scales had good convergent validity.

**Table 2.** Model specification.

Model	*X^2^*	*df*	SRMR	CFI	NFI	TLI	RMSEA
1. 6 -factor model	1455.57	573	0.054	0.958	0.927	0.953	0.048
2. 4-factor model (stigmatisation variables combined)	1327.213	577	0.042	0.957	0.926	0.953	0.052
3. 1-fator model (all variables combined)	1351.275	582	0.063	0.956	0.925	0.952	0.048

Abbreviations: *X*^2^ chi-square; *df,* degrees of freedom; SRMR, standardised root mean square residual; CFI, comparative fit index; NFI, normed fit index; TLI, Tucker –Lewis incremental fit index; RMSEA, root mean square residual.

### Correlations, mean and standard deviation analysis

The inter-factor correlation factor, mean and standard deviation analysis of all the elements are presented in Table 2. To check the discriminant validity of the scales, we assessed the latent variable correlations and the square root of the AVEs. Discriminant validity is valid under the rule of thumb when the square root of the AVE is greater than the associated correlation coefficient value.

In contrast, discriminant validity is not attained if the square root of the AVE presented along the diagonal line in the correlation matrix is less than its corresponding coefficients. Also, the square root of the AVEs, which are italicised in bold and presented along the diagonal line in the correlation matrix, are more significant than their corresponding correlation coefficients. These results show that discriminant validity has been achieved. Although the variables are related, they are distinct from each other (Table 3).

**Table 3.** Correlation analysis, discriminant validity, means and standard deviations.

	Sex	Age	Edu	Jobtype	Marr	CDP	PES	RESI	NEX	ANX	PER	Mean	Std. Dev.
Sex												1.38	0.49
Age	0.01											3.09	0.92
Edu	−.120**	0.03										2.05	0.95
Jobtype	−0.03	0.01	0.04									2.65	0.74
Mar	.14**	0.02	−0.27	0.01								1.35	0.48
CDP	−0.02	0.07	−0.03	0.08	0.01	***0.798***						4.74	0.80
PES	−0.01	−0.05	−0.04	−0.03	−0.02	−0.19	***0.99***					17.02	7.27
RESI	−0.01	−0.02	0.01	−0.03	−0.07	−0.18	.19**	***0.913***				28.91	13.50
NEX	0.01	0.05	−0.02	.13**	0.02	.56**	−0.15	−0.15	***0.763***			8.28	2.37
ANX	−0.12	0.06	0.07	0.03	0.01	.47**	−0.13	−0.17	.35**	***0.774***		13.56	3.80
PER	−0.01	−0.17	0.01	−0.09	−.087*	−0.58	.16**	.21**	−0.53	−0.52	***0.808***	8.44	3.26

*Note.* Discriminant validity values are presented in bold along with the inter-factor correlation matrix. Abbreviations: RESI, Resilience; PES, Personalised stigma; CDP, Concern for disclosure and public attitude; PER, Performance; ANX, Anxiety; and NEX, negative experience.

### Hypotheses testing

#### Hypothesis testing the main effects of COVID-19 stigmatisation and the mediating effects of anxiety in the relationship between COVID-19 stigmatisation and job performance

The study employed the hierarchical regression method to assess the hypothesised relationships while controlling for sex, age, education, job type, and marital status. Before performing the mediation analysis, the study regressed the control variables, PES, CDP, and NEX, on job performance among frontline health workers, as shown in Table 4, Models 1, 2, 3, 4, and 5. Interestingly, PES and CDP significantly impacted job performance, whereas NEX showed an insignificant negative effect. Drawing on the Golfman theory of stigmatisation, a possible explanation for this finding could be that when frontline health workers experience a high degree of COVID-19 stigmatisation than they can handle, they can become tensed and emotionally disturbed, which can compromise their job performance. Thus, it is critical to ending stigma and other inhumane actions directed toward frontline health workers who handle COVID-19 patients. The current study's findings corroborate those of [72,73], in a cross-sectional study, established that stigma can have more severe negative repercussions on workers' outcomes and performance.

**Table 4.** Hierarchical regression results of the mediating effects of anxiety in the relationship between PES, CDP, NEX, and performance.

Var	Perf. β (t)	Perf. β (t)	ANX β (t)	Perf. β (t)	Perf. β (t)
	Model 1	Model 2	Model 3	Model 4	Model 5
**Constant**	12.176*** (13.611)	.041 (.193)	−.902*** (−3.246)	17.969*** (21.237)	23.001*** (26.448)
Gender	.025 (.092)	−.431*** (−3.932)	.072 (.495)	−.383 (−1.666)	−.183 (−.913)
Edu	−.035 (−.247)	−.095 (−.684)	−.156 (−.857	.078 (.648)	−.028 (−.265)
Jobtype	−.404	−.566** (−2.553)	.281 (.960)	−.351** (−2.362)	−.133 (−1.020)
Marriage	−.589** (−2.304)	−.083 (−.979)	.102 (.960)	−.452 (−1.877)	−.496** (−2.3)
PES		−1.580 *** (−10.354)	.298*** (3.707)		−.058 (−.721)
CDP		−.416 *** (−10.354)	.309*** (4.539)		−.380** (−2.98)
NEX		.041 (.193)	.902*** (3.246)		−.339*** (−6.84)
**ANX**				−.450*** (−15.436)	−.24*** (−8.51)
R square	0.37	0.39	0.27	0.39	0.29
F	5.23	5.34	4.23	4.65	3.68

Abbreviations: Edu, Education; PES, Personalised stigma; CDP, Concern for disclosure and public attitude; NEX, negative experience ANX, Anxiety; and PER, Performance; *** and ***represent 1% and 5% levels of significance.

FHW's experience with COVID-19 stigma was significantly associated with their anxiety, consistent with previous research [74]. Earlier research [75] has discovered that stigma is associated with increased stress and poor physical health. Further, the study's findings established that anxiety negatively affects frontline health workers' job performance. The observed relationship between anxiety and job performance can be explained by the fact that highly anxious people are more focussed on reducing their anxieties and vulnerabilities than performing the task with greater interest, involvement, and zeal. Additionally, the employee with high anxiety levels often lacks self-confidence and is fearful about his ability to carry out job responsibilities, leading to self-abasement and incompetence. The current study's findings corroborate previous findings from Jones et al. [76], which revealed that work-related anxiety had been negatively linked with several workplace performance indicators. The study's conclusions again agree with the findings of Jeske et al. [77], which asserted that anxiety decreases job performance among the British and Americans.

Furthermore, in making anxiety the dependent variable in Model 3, all the stigmatisation variables (PES, CDP, and NEX) similarly had a significant positive impact on anxiety. The result, therefore, supported H2a and H2b and H2c. Finally, in model 5, we regressed PES, CDP, NEX, and anxiety on performance. The outcome showed that PES, CDP, NEX, and anxiety significantly negatively affected performance. An indication that the connections between PES, CDP, NEX, and performance were partially mediated by anxiety and, therefore, partially offered support for H3a, H3b, and H3c. The study contributed to the literature by considering anxiety as one of the conduits through which COVID-19 stigmatisation can predict employee work performance. Table summary of the hypothesis of the study is summarised in Table 5.

**Table 5.** Summary of hypotheses.

Hypotheses	Path	Remarks
H1		
H1a	PES —> JP	Supported
H1b	CDP —> JP	Supported
H1c	NEX —> JP	Supported
H2		
H2a	PES —> ANX	Supported
H2b	CDP —>ANX	Supported
H2c	NEX —> ANX	Supported
H3		
H3a	PES —> ANX–>JB	Supported
H3b	CDP —>ANX–>JB	Supported
H3c	NEX —> ANX–>JB	Supported
H4		
H4a	PES *RES–>JB	Not Supported
H4b	CDP *RES–>JB	Not Supported
H4c	NEX *RES–>JB	Supported

Abbreviations: PES, Personalised stigma; CDP, Concern for disclosure and public attitude; NEX, negative experience ANX, Anxiety; and PER, Performance.

#### Hypothesis assessing resilience's main and moderating effects on the relationship between COVID-19 stigmatisation and anxiety

**Figure 2.**
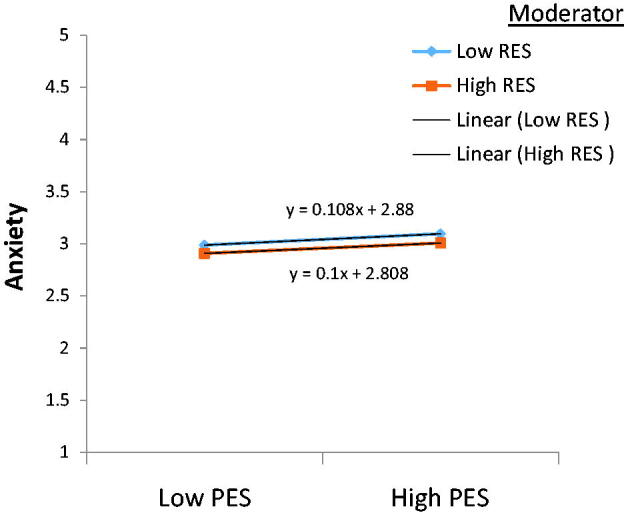
Moderating influence of resilience in the relationship between personalised stigma and anxiety.

As presented in Table 6 and Figure 2, PES significantly and positively (*β* = .053, *p* < .05). Sadly, the interaction between PES and resilience showed no statistically significant effect on anxiety, which failed to support hypothesis 4(a). By treating concern for disclosure and public attitude (CDP) as an independent variable in Table 7 and resilience as a moderating variable, we entered them into the third block to create model 3.

**Table 6.** Hierarchical regression results of the moderating role of resilience in the relationship between personalised stigma and anxiety.

Variables	Anxiety β (t)	Anxiety β (t)	Anxiety β (t)
	Model 1	Model 2	Model 3
(Const)	2.259*** (12.558)	2.838*** (13.279)	2.836 9*** (13.275)
Edu	.302 (1.781)	.271 (13.279)	.287 (1.712)
Jobtype	.133 (.638)	.098 (.479)	.092 (.447)
Sex	.016 (.106)	−.071 (−.465)	−.073 (−.479)
Marriage	.209 (.622)	.088 (.266)	.101 (.307)
PES		.053 ** (2.483)	.052** (2.457)
RESI		−.044*** (−3.805)	−.042*** (−3.862)
PES* RESI			−.002 (−.887)
R square	0.31	0.36	0.32
F	3.81	3.55	3.94

Abbreviations: Edu, Education; RESI, Resilience; PES, Personalised stigma; *** and ***represent 1% and 5% level of significance.

**Table 7.** Hierarchical regression results of the moderating impacts of resilience in the relationship between Concern for disclosure and public attitude and anxiety.

Variables	Anxiety β (t)	Anxiety β (t)	Anxiety β (t)	Decision
	Model 1	Model 2	Model 3	
(Constant)	12.259*** (12.558)	16.084*** (17.753)	3.344*** (8.484)	
Edu	.302 (1.781)	.347** (2.328)	.342** (2.300)	
Jobtype	.133 (.638)	−.054 (−.294)	−.074 (−.402)	
Sex	.016 (.106)	−.149 (−1.098)	−.174 (−1.279)	
Marriage	.209 (.622)	.128 (.432)	.093 (.316)	
CDP		.431*** (12.481)	.421*** (12.602)	
RESI		−.025** (−2.484)	−.024** (−.418)	
CDP* RESI			−.025 (−3.166)	
R square	0.314	0.312	0.318	
F	4.21	5.53	6.84	

Abbreviations: Edu, Education; RESI, Resilience; CDP, Concern for disclosure and public attitude; *** and ***represent 1% and 5% significance level.

It can be deduced from Table 7 that concern for disclosure and public attitude negatively predicted anxiety. Concern for disclosure and public attitude influenced anxiety by *β* = 0.421, *p* < .01, whereas resilience showed a negative sign with *β* = −.024, *p* < .01. Interestingly, the interactive effect (CDP × resilience) showed an insignificant negative sign on anxiety, failing to support H4b.

The next section of the study entered the control variables, negative experiences, and resilience in the regression equation to predict anxiety. As shown in Table 8, there is a significant positive influence of negative experiences on anxiety, while resilience showed a significant negative effect.

**Table 8.** Hierarchical regression results of the moderating influence of resilience in the relationship between negative experience and anxiety.

Variables	Anxiety β (t)	Anxiety β (t)	Anxiety β (t)
	Model 1	Model 2	Model 3
(Constant)	2.259*** (12.558)	3.230 *** (14.715)	3.421*** (4.999)
Edu	.302 (1.781)	.330** (2.129)	.316 ** (2.050)
Jobtype	.133 (.638)	−.131 (−.678)	−.127 (−.663)
Tenure	.016 (.106)	−.050 (−.356)	−.084 (−.598)
Marriage	.209 (.622)	.111 (.361)	.106 (.347)
NEX		.597*** (9.841)	.400*** (9.955)
RESI		−.033 *** (−3.118)	−.041*** (−3.793)
NEX* RESI			−.012** (−3.121)
R square	0.42	0.27	0.39
F	4.12	4.18	3.84

Abbreviations: Edu, Education; RESI, Resilience; NEX, negative experience*** and ***represent 1% and 5% significance level.

**Figure 3.**
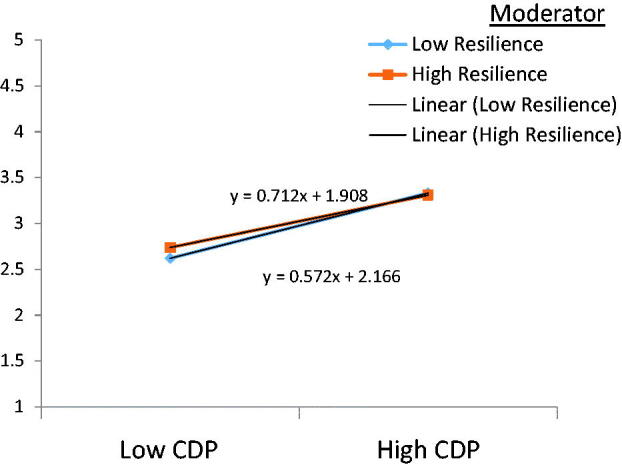
Moderating impacts of resilience in the relationship between concern for disclosure and public attitude (CDP) and anxiety.

**Figure 4.**
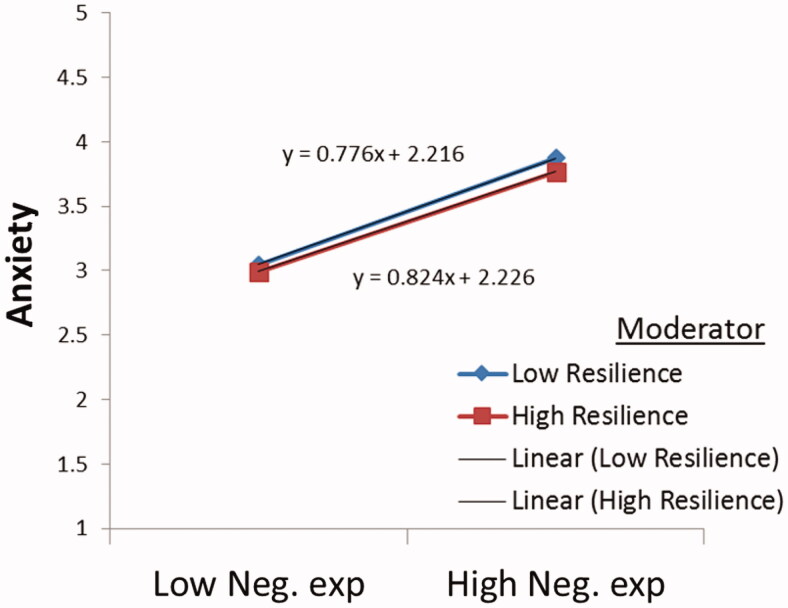
Moderating influence of resilience in the relationship between negative experience and anxiety.

Not only was the direct connection between resilience and anxiety confirmed, but our study also established for the first time the moderating effect of resilience on the relationship between negative experiences from COVID-19 stigmatisation (except for personalised stigma and concern for disclosure and public attitude) and anxiety through a simple slope analysis. The results from Tables 6–8 and Figures 2–4 show that an increased resilience level though insignificant reduces the positive relationship between personalised stigma and anxiety (*t* = .07, *p* = .95). Similarly, when resilience is high, the relationship between concern for disclosure and public attitude and anxiety is dampened and insignificant. Resilience could not explain the persisting association between personalised stigma, concern for disclosure, and public attitude and anxiety, which possibly reflects the study’s limitation. This can also be because we could only collect the data in three different waves, with a one-month data collection gap. Perhaps resilience would have moderated the relationship between personalised stigma, concern for disclosure and public attitude, and anxiety had the data been collected in more than three different waves.

Unlike personalised stigma and concern for disclosure and public attitude, the predictive effects of negative experience on anxiety are reduced and significant with the increased resilience level. This means that resilience could serve as a defensive factor buffering the negative experience of COVID-19 stigmatisation on anxiety. The current study's findings support those of Ong et al. [78], who discovered that individuals with greater resilience have a greater capacity to overcome obstacles in life.

### Limitations of the study

While this study makes an essential contribution to human resources and the perspective of health management, it has some limitations to be considered in upcoming research.

- The first approach for collecting data was a lag of one month. Although this strategy gives some evidence of the time nexus, the causal effect compared to the longitudinal study approach is restricted. Future research should collect data at longer intervals, as suggested by Grandey et al. [79]. A larger sample and multiple hospitals in Ghana and worldwide are recommended to generalise and cross-validate the existing results.
- Second, stigma is a multi-faceted concept with some aspects that are not measured; for example, there has been a focus on implicit stigma measures [80]. Future studies should explore the effects of the other aspects of the stigma that were not considered in this study on health workers' performance.

## Conclusion and policy implication

COVID-19 stigmatisation is a significant problem in Ghana and requires urgent attention from policymakers. This paper aims to examine the effect of COVID-19 stigmatisation on job performance among frontline health workers. Based on Goffman's theory, the study contributes to the literature on stigmatisation by exploring: a. the mediating role of anxiety in the link between COVID-19 stigmatisation and job performance, b. the moderating effects of resilience in the association between COVID-19 stigma and anxiety among frontline health workers. In conclusion, our study demonstrates that COVID-19 stigmatisation among frontline health workers directly affects their job performance. The findings further found that anxiety partially mediated the association between COVID-19 stigmatisation (Concern for disclosure and public attitude and negative experience) and job performance, whereas personalised stigma was insignificant. The present study's findings again elucidate that resilience moderated the connection between only one component of COVID-19 stigmatisation (negative experience) and anxiety.

Despite the current study's limitations, the findings have substantial clinical relevance. Since COVID-19 does not discriminate against anyone, it is critical to combat stigma at the community and individual levels to mitigate its adverse impacts on job performance. Cognitive-behavioral therapy (CBT) is recommended as the first line of psychological treatment for health workers afflicted with COVID-19 stigma. The origin of CBT therapies for stigmatisation has incorporated a normalising rationale to diminish the people’s experience of being distinct from others [81]. Providing information regarding the general occurrence of COVID-19 stigma experiences should be part of normalisation strategies. CBT has been shown to boost self-esteem in individuals who feel stigmatised [82].

Not only that, ensuring that stigmatising and discriminatory behaviours are recognised and eliminated as part of COVID-19 the national preventive and response strategies. This will help combat hostility and hateful speech aimed directly at health workers to safeguard mental health and job performance in times of natural disasters. The current study found resilience to moderate the link between COVID-19 stigmatisation (Concern for disclosure and public attitude and negative experience) and anxiety. The improvement of resilience could thus be a new insight in reducing anxiety among health workers who experience stigmatisation of COVID-19.

Finally, we described how anxiety mediates the association between the stigmatisation of COVID-19 and the performance of health care providers. We hope that our results will help better understand the links between anxiety, COVID-19 stigma, and performance and will guide the development of effective measures to improve the performance of health care providers on the front lines.

## Data Availability

Data is available upon reasonable request from the corresponding author.
